# Supramolecular Phase-Selective Gelation by Peptides Bearing Side-Chain Azobenzenes: Effect of Ultrasound and Potential for Dye Removal and Oil Spill Remediation

**DOI:** 10.3390/ijms160511766

**Published:** 2015-05-22

**Authors:** Jürgen Bachl, Stefan Oehm, Judith Mayr, Carlos Cativiela, José Juan Marrero-Tellado, David Díaz Díaz

**Affiliations:** 1 Institut für Organische Chemie, Universität Regensburg, Universitätsstr. 31, Regensburg 93053, Germany; E-Mails: bachl_j@web.de (J.B.); stefan.oehm@tu-berlin.de (S.O.); judith.mayr@chemie.uni-regensburg.de (J.M.); 2 Instituto de Síntesis Química y Catálisis Homogénea (ISQCH), CSIC-University of Zaragoza, 50009 Zaragoza, Spain; E-Mail: cativiela@unizar.es; 3 Departamento de Química Orgánica, IUBO, “Antonio González”, Universidad de La Laguna, Astrofísico Francisco Sánchez 2, 38206 La Laguna, Spain; E-Mail: jtellado@ull.es; 4 IQAC-CSIC, Jordi Girona 18-26, 08034 Barcelona, Spain

**Keywords:** peptide, azobenzene, side-chain functionalization, organogel, phase-selective gelation, dye removal, oil spill

## Abstract

Phase selective gelation (PSG) of organic phases from their non-miscible mixtures with water was achieved using tetrapeptides bearing a side-chain azobenzene moiety. The presence of the chromophore allowed PSG at the same concentration as the minimum gelation concentration (MGC) necessary to obtain the gels in pure organic phases. Remarkably, the presence of the water phase during PSG did not impact the thermal, mechanical, and morphological properties of the corresponding organogels. In the case of miscible oil/water mixtures, the entire mixture was gelled, resulting in the formation of quasi-hydrogels. Importantly, PSG could be triggered at room temperature by ultrasound treatment of the mixture or by adding ultrasound-aided concentrated solution of the peptide in an oil-phase to a mixture of the same oil and water. Moreover, the PSG was not affected by the presence of salts or impurities existing in water from natural sources. The process could be scaled-up, and the oil phases (e.g., aromatic solvents, gasoline, diesel fuel) recovered almost quantitatively after a simple distillation process, which also allowed the recovery and reuse of the gelator. Finally, these peptidic gelators could be used to quantitatively remove toxic dyes from aqueous solutions.

## 1. Introduction

Among numerous synthetic stimuli-responsive materials, supramolecular (or physical) gels [1,2,3,4,5,6,7,8] have been recognized as promising materials for high-tech applications in important areas such as catalysis, biomedicine, cosmetics, foods, sensors, and environmental remediation [3,9–13]. In contrast to chemical gels [14,15,16], which are based on covalent bonds, supramolecular gels are made of either low-molecular-weight (LMW) compounds or polymers that are self-assembled by multiple non-covalent interactions (e.g., hydrogen-bonding, van der Waals, charge-transfer, donor-acceptor, dipole-dipole, π–π stacking, coordination interactions). The solid-like appearance of these fascinating materials is the result of the entrapment of the liquid (major component) into the interstices of a self-assembled matrix of large surface area (minor component), a process that is typically induced by a heating-cooling treatment of the mixture. Due to the weakness of the non-covalent interactions present in the network structure, supramolecular gels are usually thermoreversible.

On the other hand, despite the large number of LMW organogelators described in the literature so far, only a minor fraction have shown the ability to gel one solvent in preference to another from a given mixture, particularly if one of the solvents is water that could cause a major disruption of hydrogen-bonded networks [17,18,19,20,21,22,23,24,25,26,27,28,29]. Within this context, phase-selective gelation (PSG) of organic oils from aqueous mixtures is being considered a promising alternative to bioremediation [30] to allow the removal and reuse of oils from spills, which are associated with irreversible environmental damages especially in marine ecosystems. Other materials used in oil spill recovery include solidifiers [31], dispersants [32], and sorbents [33,34,35,36]. However, there are practical limitations concerning the use of thermal treatments of flammable mixtures, confining the oil spill, and oil recovery using traditional methods. In 2001, Bhattacharya and Krishnan-Ghosh reported the first phase selective gelator based on an alanine amphiphile [37]. Since then several other groups have reported very interesting LMW gelators with PSG ability [17,18,19,20,21,22,23,24,25,26]. The main motivation for this development has been the necessity of finding (1) more efficient methods for applying the gelator; (2) new approaches to induce gelation at room temperature (e.g., mechanical shaking [21,22], sonication [19], addition of predissolved gelator in a cosolvent [19,25,26,27,28,29]); and (3) more robust stimuli-responsive gel materials that allow the recovery of both the oil phase and the gelator for further use [24,25,26,28,29].

Within this context, and as part of our main research program devoted to the development of new soft gel-based materials for different applications, we have recently reported a new series of multistimuli-responsive supramolecular gels using for the first time LMW peptides bearing side-chain azobenzene moieties [38]. Interestingly, the azobenzene residue can be used as a versatile regulator to reduce the critical gelation concentration and enhance both the thermal stability and mechanical strength of the gels. The major driving forces for the gelation process are hydrogen-bonding and π–π interactions, which can be triggered either by thermal or ultrasound external stimuli, affording materials having virtually the same properties.

**Figure 1:**
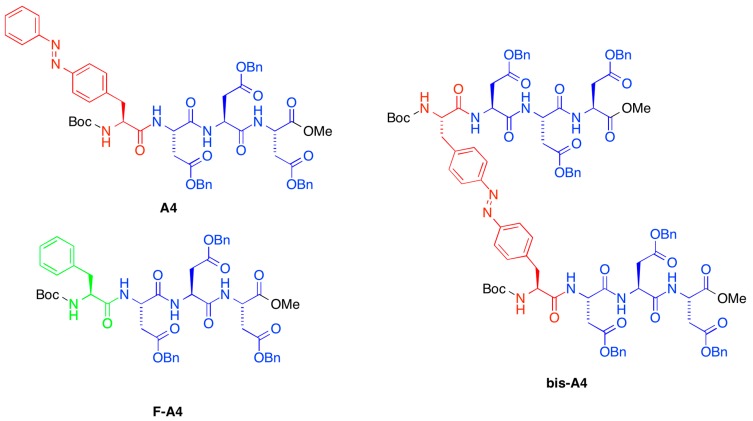
Structure of peptidic gelators used in this work (Bn = CH_2_Ph).

Herein, we report the discovery of selective and quantitative gelation of organic phases from oil/aqueous mixtures using model tetrapeptide-based gelators (Figure 1) that we described earlier [38]. Moreover, these materials could also be used for the uptake of toxic dyes from aqueous solutions.

## 2. Results and Discussion

### 2.1. Synthesis

**Scheme 1:**
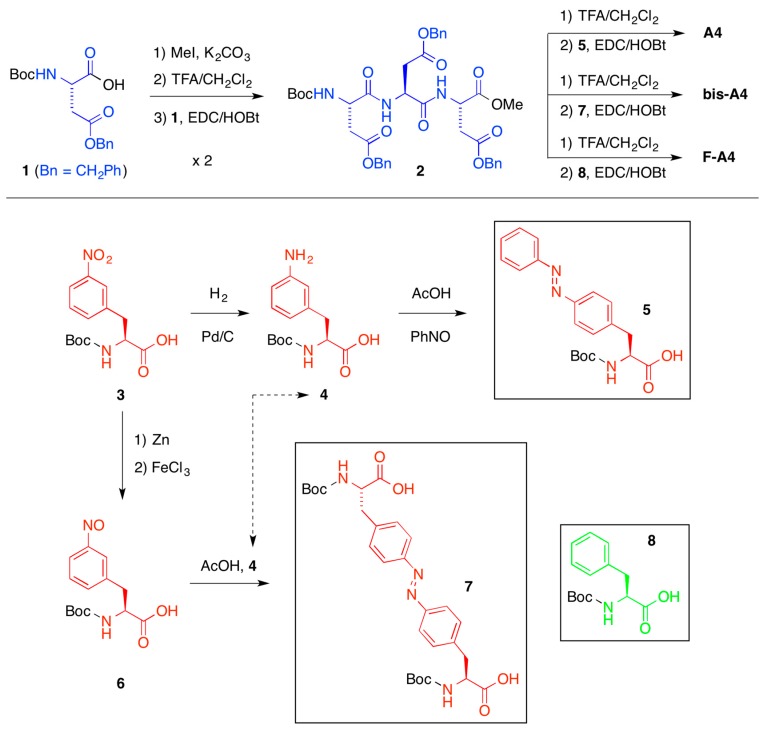
General synthesis of tetrapeptides **A4**, **bis-A4**, and **F-A4** (EDC = *N*-(3-dimethylaminopropyl)-*N'*-ethylcarbodiimide hydrochloride; HOBt = 1-hydroxybenzotriazole); TFA = trifluoroacetic acid).

The synthesis of the tetrapeptides used in this work (Figure 1) was carried out in solution employing standard EDC/HOBt as coupling agents and following the experimental procedures previously reported [38]. In brief, required *C*-terminal methyl esters **2** (Scheme 1, *top*) were prepared by simple reaction of the corresponding *N*-Boc-protected amino acid **1** with MeI. Boc deprotection and subsequent coupling with the suitable *N*-Boc protected azobenzene-based amino acid (*i.e.*, Boc-azoPhe-OH (**5**) and Boc-bisazoPhe (**7**)) or Boc-Phe-OH (**8**) afforded the desired tetrapeptides after purification by column chromatography. Key building blocks **5** and **7** were rapidly synthesized starting from commercial *N*-Boc-(*p*-nitro)-l-phenylalanine (**3**), where the crucial step was the condensation of suitable amino derivative **4** and nitroso compounds (**6** or PhNO) under acidic conditions (Scheme 1, *bottom*).

### 2.2. Phase-Selective Gelation (PSG)

A preliminary screening of the peptide library synthesized in our previous work [38] showed a significant ability to selectively gel organic solvents from their mixtures with water after heating-cooling treatment. In these experiments the water phase remained in the fluid state. Due to very similar physical properties of the gels observed within each group [38,39], PSG of oil/water mixtures (1:2 *v*/*v*) was studied using only some representative compounds, *i.e.*, **A4**, **bis-A4**, **F-A4**, as shown in Figure 1. Very interestingly, we found that the presence of side-chain azobenzene moieties in the gelator structure (**A4**, **bis-A4**) allowed PSG of the oil phase within minutes at the same concentration as the described minimum gelation concentration (MGC) for pure organic phases (POP) [38] (Table 1, entries 1–14). In contrast, the use of Phe-based gelators (**F-A4**) required *ca.* two times higher concentration than the corresponding MGC in POP (Table 1, entries 15–21). Therefore, we decided to continue further studies with **A4** and **bis-A4** as model systems, which also showed the ability to gel ionic liquids (*i.e.*, 1-butyl-3-methylimidazolium hexafluorophosphate, BMIM·PF_6_) either as pure solvents or in aqueous mixtures.

**Table 1:** Comparison of the gelation properties of pure organic phases (POP) and phase-selective gelation (PSG) for selected organic phases.

Entry	Organic Phase	Gelator	Minimum Gelation Concentration, MGC (% *w*/*v*)		Gel-to-Sol Transition Temperature, *T*_gel_ (°C)	
			POP	PSG	POP	PSG
1	2-BuOH	**A4**	2.8 ± 0.3	3.0 ± 0.4	55 ± 1	54 ± 2
2	BMIM·PF_6_	**A4**	4.0 ± 0.3	4.0 ± 0.3	78 ± 2	76 ± 1
3	Toluene	**A4**	1.9 ± 0.2	2.0 ± 0.2	37 ± 2	39 ± 1
4	Xylene	**A4**	7.0 ± 0.5	7.0 ± 0.5	59 ± 1	62 ± 2
5	Olive Oil	**A4**	1.3 ± 0.2	1.5 ± 0.2	93 ± 2	89 ± 1
6	Gasoline	**A4**	2.2 ± 0.2	2.4 ± 0.2	52 ± 2	53 ± 2
7	Diesel	**A4**	2.0 ± 0.2	2.0 ± 0.2	62 ± 1	61 ± 1
8	2-BuOH	**Bis-A4**	0.8 ± 0.1	1.0 ± 0.1	62 ± 2	63 ± 1
9	BMIM·PF_6_	**Bis-A4**	2.2 ± 0.2	2.2 ± 0.2	69 ± 1	68 ± 2
10	Toluene	**Bis-A4**	0.6 ± 0.1	0.5 ± 0.1	70 ± 1	70 ± 2
11	Xylene	**Bis-A4**	3.3 ± 0.2	3.5 ± 0.3	76 ± 2	74 ± 2
12	Olive Oil	**Bis-A4**	0.5 ± 0.1	0.5 ± 0.1	104 ± 2	101 ± 2
13	Gasoline	**Bis-A4**	2.5 ± 0.2	2.5 ± 0.2	56 ± 1	55 ± 2
14	Diesel	**Bis-A4**	1.8 ± 0.2	2.0 ± 0.2	59 ± 2	61 ± 1
15	2-BuOH	**F-A4**	10.0 ± 1.0	13.0 ± 1.5	57 ± 1	52 ± 2
16	BMIM·PF_6_	**F-A4**	-	-	-	-
17	Toluene	**F-A4**	2.5 ± 0.3	4.4 ± 0.3	35 ± 1	44 ± 1
18	Xylene	**F-A4**	2.0 ± 0.2	3.2 ± 0.3	37 ± 1	36 ± 2
19	Olive Oil	**F-A4**	2.0 ± 0.2	2.8 ± 0.3	72 ± 2	71 ± 1
20	Gasoline	**F-A4**	3.3 ± 0.3	4.5 ± 0.5	54 ± 2	57 ± 2
21	Diesel	**F-A4**	1.8 ± 0.2	2.4 ± 0.2	56 ± 2	54 ± 1

**Figure 2:**
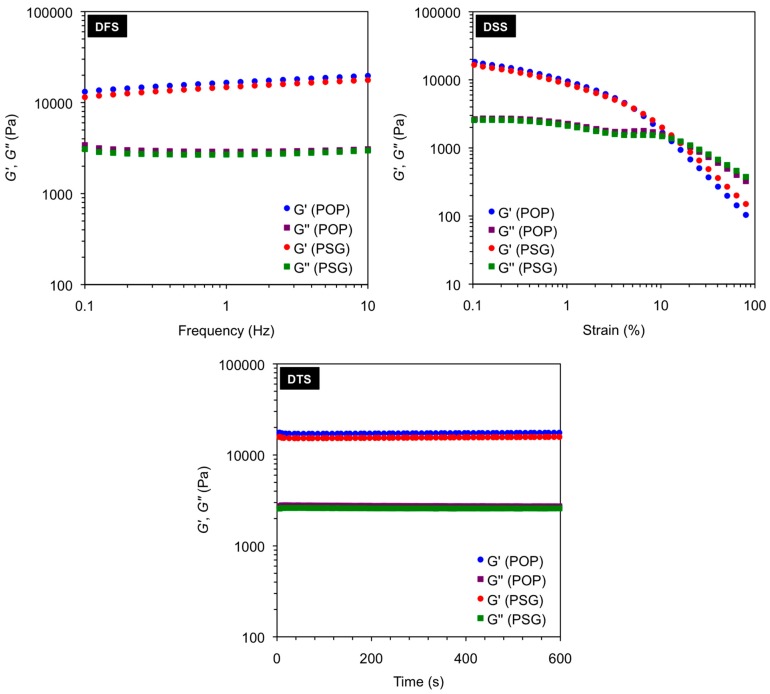
Dynamic oscillatory rheological measurements (*i.e.*, DFS, DSS, DTS) of the gels in toluene obtained after gelation of pure organic phase (POP) and phase selective gelation (PSG) of toluene/water mixture (1:2 *v*/*v*) using **A4** as gelator (2.0% *w*/*v*). Heating-cooling treatment was used in these examples to induce gelation. Average storage modulus *G'* (16.5 ± 1.97 kPa for POP; 14.7 ± 1.89 kPa for PSG), average loss modulus *G''* (3.0 ± 0.12 kPa for POP; 2.8 ± 0.11 kPa for PSG), average tan δ (0.18 ± 0.0028 for POP; 0.19 ± 0.0019 for PSG), and maximum strain at break (16% ± 2.0% for POP; 13% ± 1.5% for PSG) remained constant within the limits of the experimental error regardless the material. As observed in POP, the gels obtained from PSG displayed *G'* at least one order of magnitude higher than *G''* and relatively low dependence with frequency, indicating the viscoelastic nature of the samples as well as a good tolerance to external forces.

**Figure 3:**
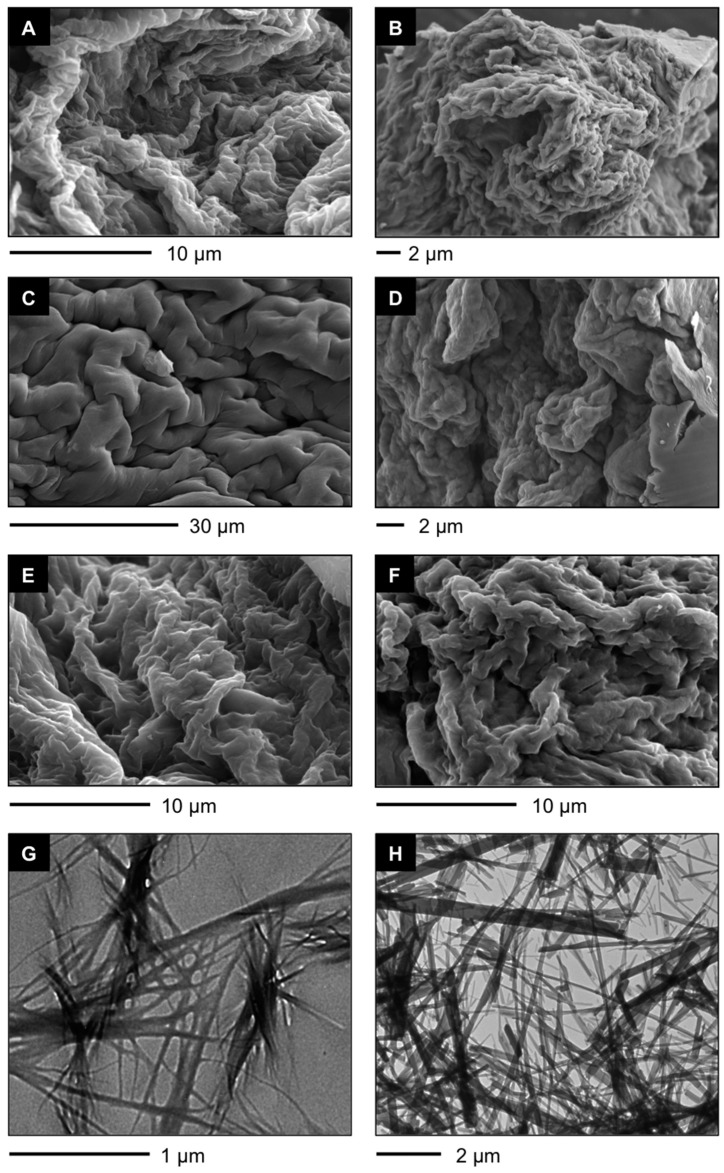
(**A**–**F**) Representative field emission scanning electron microscope (FESEM) images of xerogels prepared by freeze-drying the corresponding gel materials obtained at the minimum gelation concentration (MGC) as indicated in Table 1: (**A**) **A4** in toluene (POP) [38]; (**B**) **A4** in toluene (PSG); (**C**) **F-A4** in toluene (POP) [38]; (**D**) **F-A4** in toluene (PSG); (**E**) **Bis-A4** in toluene (POP) [38]; (**F**) **Bis-A4** in toluene (PSG); and (**G**,**H**) Representative transmission electron microscope (TEM) images of xerogels prepared by freeze-drying the corresponding gel materials obtained at the MGC as indicated in Table 1; (**G**) **A4** in toluene (POP) [38]; (**H**) **A4** in toluene (PSG).

It should be emphasized that many other solvents previously reported to be gelled by the different peptides could also be selectively gelled from their mixtures with water [38,39]. Remarkably, the presence of the water phase during PSG did not cause a significant impact on the thermal, mechanical, and morphological properties of the corresponding organogels bodies as determined by *T*_gel_ measurements (Table 1), dynamic rheological measurements (Figure 2) and electron microscopy (Figure 3), respectively. Thus, the presence of water during PSG does not disrupt (e.g., by partial diffusion to the organic layer) the pattern of non-covalent interactions responsible for the gelation of the oil phase [38]. In water, the peptidic gelator excludes water due a hydrophobic effect caused by the presence of lipophilic moieties (an effect which is larger in **A4** than in **F-A4** due to the presence of side-chain azobenzene units, which could explain the differences observed on the MGC). This gives room for the self-assembly of the gelator in the oil phase driven by non-covalent interactions such as hydrogen-bonding, π–π stacking and/or van der Waals [38]. However, further studies are still necessary to determine the exact conformation of these peptides in hydrophobic environments, and quantify the interactions that are responsible for the gelation process.

**Figure 4:**
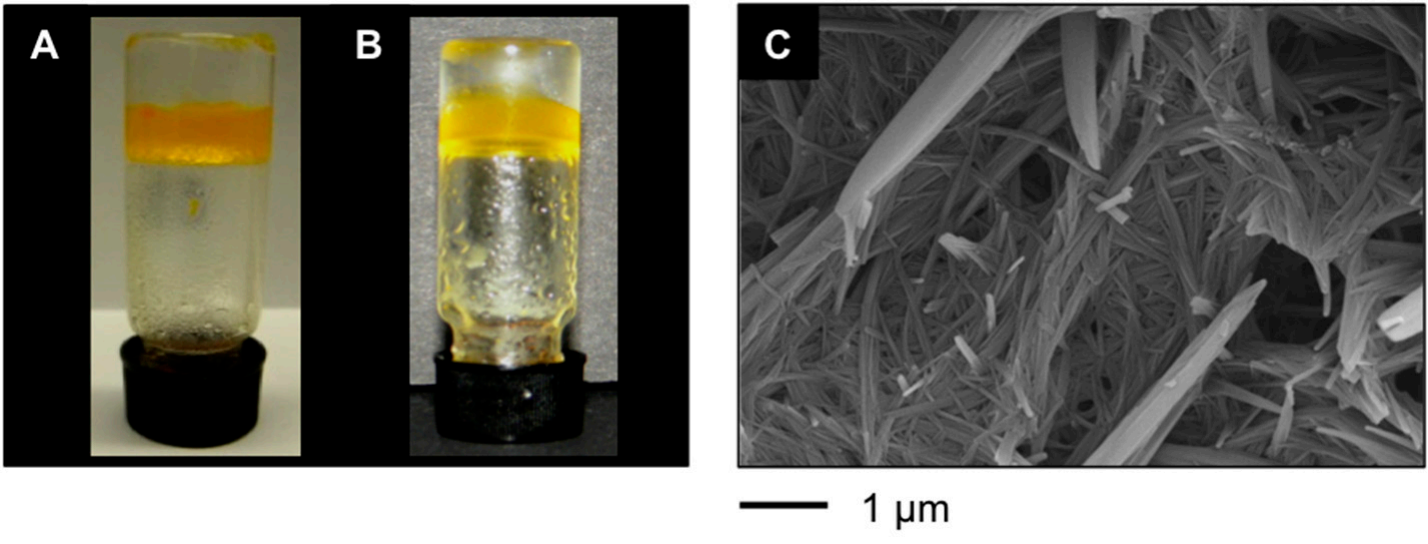
(**A**) Typical appearance of an inverted vial containing PSG of a mixture xylene/water prepared with **A4** as shown in Table 1; (**B**) PSG of gasoline/water mixture; and (**C**) FESEM image of the xerogel prepared by freeze-drying the gel obtained by PSG of a mixture of gasoline/water (1:2 *v*/*v*) using **bis-A4** as shown in Table 1.

Additionally, our LMW peptidic gelators also allowed PSG of environmental hazardous materials including gasoline and diesel fuel (Figure 4A,B), affording gel phases that were characterized by fibrillar networks as shown in Figure 4C.

**Figure 5:**
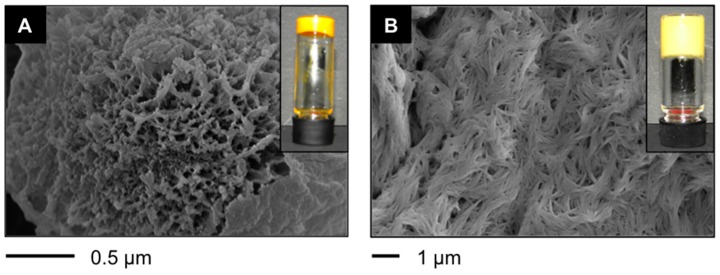
(**A**) FESEM image of the xerogel obtained from the corresponding organogel made in *i*-PrOH using **A4** (2.0% *w*/*v*) (inset: photograph of the gel) [38]; and (**B**) FESEM image of the xerogel obtained from the corresponding organogel made in *i*-PrOH/water mixture (1:2 *v*/*v*) using **A4** (0.7% *w*/*v*) (inset: photograph of the gel).

Encouraged by these results, we also investigated the more challenging PSG of water-miscible organic solvents (*i.e.*, CH_3_CN, EtOH, *i*-PrOH) where gelation of the POP was previously observed [38]. Surprisingly, we found gelation of the entire organic solvent/water mixture (1:2 *v*/*v*) resulting in the formation of quasi-hydrogels with a total content of organic phase of 33% (*v*/*v*) and different morphological features in comparison to the organogels prepared in pure organic solvents. For instance, the organogel obtained in *i*-PrOH was characterized by spherical structures with a fibrillar porous inner part [38], whereas the material obtained from *i*-PrOH/water mixture revealed a high-density fibrillar network without globular microstructures (Figure 5). Interestingly enough, a much lower concentration of peptide was necessary to gel the mixture of solvent in comparison to the pure organic phase (*i.e.*, 0.7% *w*/*v* for *i*-PrOH/water (1:2 *v*/*v*) *versus* 2.0% *w*/*v* for pure *i*-PrOH). This opens the door for future research to minimize the oil phase content and/or the use of biocompatible oil phases, which would expand the potential of these gels for the encapsulation and controlled release of hydrophobic drugs.

One of the major drawbacks of some PSG described in the literature is the heating-cooling protocol necessary to induce gelation, which makes it impractical for the removal of oil spills due to the high flammability of most oil-phases (e.g., gasoline, diesel). Thus, taking advantage of our previous observation of ultrasound-induced gelation [38] we were able to successfully use this triggering mechanism for PSG at room temperature. Ultrasound-induced gelation, mainly by solvent cavitation and mismatched intra-/intermolecular interactions, has been recognized as a paradigm shift within the field of supramolecular gels [40]. With the aim of improving the viability of the process, we were delighted to observe that the addition of slightly warmed or ultrasound-aided concentrated solutions (20%–40% *w*/*v*) of the suitable gelator in an oil-phase to a mixture of the same oil and water resulted in very effective PSG. Importantly, no discrepancies in MGC values were observed for the different methods used to induce PSG (Table 2). The latter strategy avoids the use of other water-miscible cosolvents in order to predissolve the gelator, which could be potentially toxic and exhibit a negative impact on foreseeable practical applications.

**Table 2:** Comparison of the MGC necessary to induce PSG in selected oil/water mixtures either by the heating-cooling method (HC), ultrasound treatment of the mixture (US) or by adding an ultrasound-aided concentrated solution of the gelator in an oil-phase to the corresponding oil/water mixture (CS).

Entry	Organic Phase	Gelator	Minimum Gelation Concentration, MGC (% *w*/*v*)			
			POP	PSG (HC)	PSG (US)	PSG (CS)
1	Toluene	**A4**	1.9 ± 0.2	2.0 ± 0.2	2.0 ± 0.2	2.0 ± 0.2
2	Gasoline	**A4**	2.2 ± 0.2	2.4 ± 0.2	2.4 ± 0.2	2.4 ± 0.2
3	Diesel	**A4**	2.0 ± 0.2	2.0 ± 0.2	2.0 ± 0.2	2.0 ± 0.2
4	Toluene	**Bis-A4**	0.6 ± 0.1	0.5 ± 0.1	0.5 ± 0.1	0.5 ± 0.1
5	Gasoline	**Bis-A4**	2.5 ± 0.2	2.5 ± 0.2	2.5 ± 0.2	2.5 ± 0.2
6	Diesel	**Bis-A4**	1.8 ± 0.2	2.0 ± 0.2	2.0 ± 0.2	2.0 ±0.2

**Figure 6:**
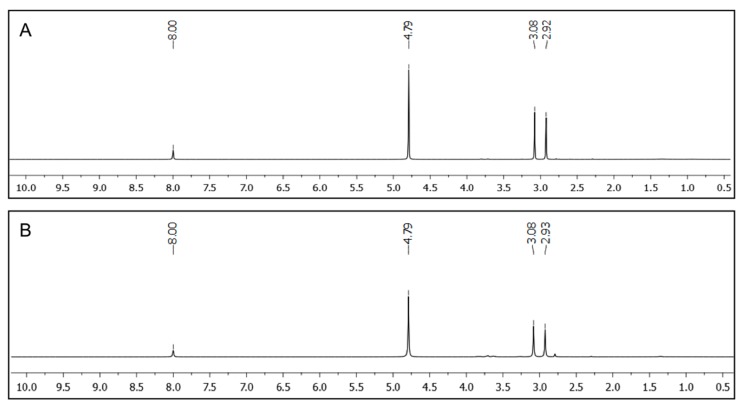
^1^H NMR spectra of the aqueous phases separated from D_2_O/toluene mixtures after PSG using **A4** as gelator. PSG was induced by the heating-cooling method and DMF (0.1 mmol) was used as internal standard. (**A**) Solvent mixture: D_2_O/toluene (2:1 *v*/*v*); [**A4**] = 2.0% *w*/*v*; (**B**) Solvent mixture: D_2_O/toluene (2:1 *v*/*v*); [**A4**] = 7.0% *w*/*v*.

The complete removal of the oil-phase from oil/water model mixtures was confirmed by ^1^H NMR spectroscopy using D_2_O as aqueous phase containing DMF as internal standard (Figure 6). No indication of signals referring to toluene or xylene could be found in the corresponding spectra when peptidic gelator **A4** (2.0% *w*/*v* and 7.0% *w*/*v*, respectively) was used for the PSG induced by the heating-cooling method. Additionally, no gelator could be detected by NMR analysis of the aqueous phase. Therefore, the term PSG is not strictly correct in these situations [18] because there is not a significant partition of the gelator into the aqueous phase. However, the term has been widely adopted to describe similar cases [17,18,19,20,21,22,23,24,25,26,27,28,29,30,31,32,33,34,35,36,37]. Furthermore, it should be considered that only a minor fraction of known non-water soluble organogelators has shown PSG ability, which makes the described tetrapeptides very interesting for both fundamental and applied sciences. 

In order to evaluate the potential application of PSG triggered by these peptides for natural oil spills, we made comparative experiments using biphasic systems containing pure water, salty water (NaCl 3.3% *w*/*v*), and natural river water (Table 3). The results showed no differences between the mixtures in terms of gelation efficiency and MGC that could be caused by the modification of the ionic strength or the presence of other impurities in natural water. These results demonstrate the robustness of the process.

**Table 3:** Comparison of the MGC necessary to induce PSG in selected oil/aqueous mixtures using different aqueous phases.

Entry	Organic Phase	Gelator	Minimum Gelation Concentration, MGC (% *w*/*v*)		
			Pure Water	NaCl 3.3% *w*/*v*	River Water
1	Toluene	**A4**	2.0 ± 0.2	2.0 ± 0.2	2.0 ± 0.2
2	Gasoline	**A4**	2.4 ± 0.2	2.4 ± 0.2	2.4 ± 0.2
3	Diesel	**A4**	2.0 ± 0.2	2.0 ± 0.2	2.0 ± 0.2
4	Toluene	**Bis-A4**	0.5 ± 0.1	0.5 ± 0.1	0.5 ± 0.1
5	Gasoline	**Bis-A4**	2.5 ± 0.2	2.5 ± 0.2	2.5 ± 0.2
6	Diesel	**Bis-A4**	2.0 ± 0.2	2.0 ± 0.2	2.0 ± 0.2

**Figure 7:**
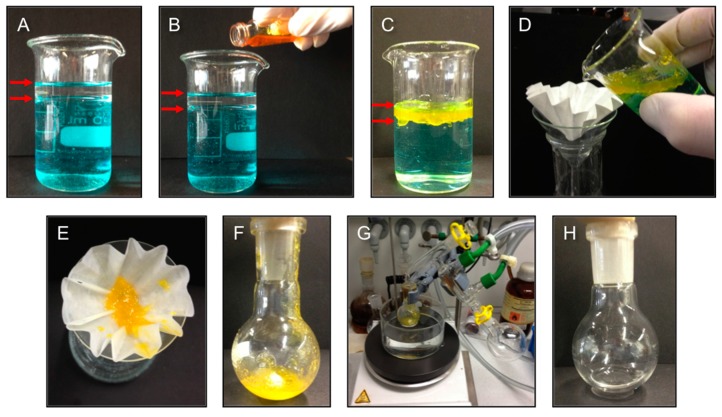
Model lab bench set up for oil spill recovery using the PSG ability of azobenzene-containing peptide **A4** (2.0% *w*/*v* with respect to the volume of the oil-phase). (**A**) Mixture of salty water (NaCl 3.3% *w*/*v*, 40 mL) and toluene (5 mL). The aqueous layer was stained with CuSO_4_ (Figure 7A) for better visualization of the two-phases indicated by red arrows; (**B**) Addition ultrasound-aided concentrated solution of gelator **A4** (27.5% *w*/*v*) in a small volume of toluene (0.4 mL); (**C**) Formation of a yellowish gel-phase containing the toluene layer; (**D**,**E**) Separation of the gel-phase by filtration; (**F**,**G**) Distillation of the gel-phase to recover both the yellowish gelator and the oil phase; and (**H**) Isolated toluene phase after distillation.

The relatively high mechanical strength of the gel-materials as well as their high temporal stability (*i.e.*, inverted vials containing the gelled oil phase could keep the weight of the aqueous phase for at least two months without any visible gravitational flow) make these LMW peptidic gelators suitable for application in oil spill recovery if the process can be scaled-up. In order to demonstrate this potential, a model scenario was set up as shown in Figure 7. Herein, toluene (5 mL) was poured into a much larger volume of salty water (NaCl 3.3% *w*/*v*, 40 mL). The aqueous layer was stained with CuSO_4_ (Figure 7A) for better visualization of the two-phase nature of the mixture (as with NaCl, the presence of the metal salt did not affect the gelation). The entire toluene-layer could be efficiently gelled by adding an ultrasound-aided concentrated solution of gelator **A4** (27.5% *w*/*v*) in a small volume of toluene (0.4 mL). This resulted in a gelator concentration of 2.0% *w*/*v* with respect to the toluene-phase (Figure 7B). Taking advantage of the thermoreversibility of the resulting supramolecular gel, which melts at *ca.* 40 °C, the gel-phase (Figure 7C) could be easily separated by filtration and subsequently distilled (Figure 7D–G) to recover both the gelator (yellowish residue that stays in the round bottom flask) and the toluene phase in almost quantitative yield (87% ± 7% in volume) (Figure 7H). The recovered gelator was reused in a new cycle with a fresh oil/water mixture at the same original concentration (2.0% *w*/*v*) affording the same yield of distilled oil. It is important to mention that gelation could be also achieved even under mechanical agitation (mimicking a real emulsion-like oil spill), albeit the gel body suffered partial fragmentation into floating gel pieces. The same experiment was also repeated using other oil-phases such as gasoline and diesel fuel with practically identical results for the oil recovery. Such efficient recovery of the oil from the gel phase and recycling of the gelator have been only reported recently on a few occasions [24,25,26].

### 2.3. Dye Removal

**Figure 8:**
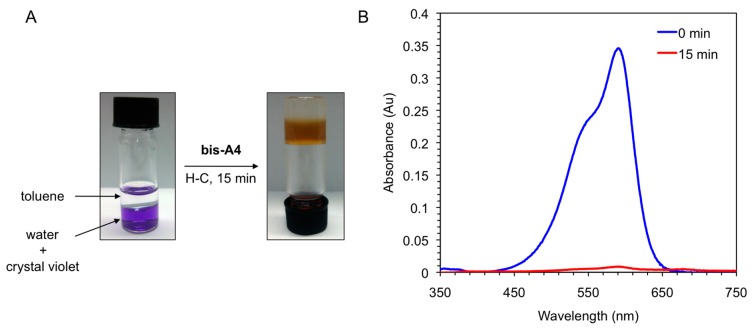
(**A**) Photographs illustrating the removal of crystal violet from an aqueous solution (*c* = 1 × 10^−5^ mol·L^−1^) by addition of toluene (left vial, water/toluene 1:1 *v*/*v*) and gelator **bis-A4** (1.0% *w*/*v*). PSG was induced by heating-cooling (H-C) treatment (right vial); and (**B**) UV-vis absorption spectra of the initial aqueous solution of crystal violet (*t* = 0 min; blue line) and of the aqueous phase after PSG (*t* = 15 min; red line).

To demonstrate another practical application of our system, we took advantage of the PSG ability of the LMW peptidic gelators to quantitatively remove water-soluble dyes [41] from aqueous mixtures within minutes (Figure 8). In two model examples, gelator **bis-A4** was used to remove crystal violet (CV) from aqueous phases. The addition of a toluene phase and the peptidic gelator at a concentration that should guarantee the complete gelation of the organic phase, caused a rapid migration of the dye from the aqueous to the organic layer with simultaneous entrapment of the dye after heating-cooling treatment of the mixture. The dye was almost quantitatively (>95%) removed from the aqueous phase within minutes as determined by UV-vis spectroscopy (Figure 8). Preliminary experiments [39] showed that other dyes such as ruthenium red could also be removed from aqueous solutions using other peptidic gelators bearing a side-chain azobenzene moiety (data not shown) [38]. 

**Figure 9:**
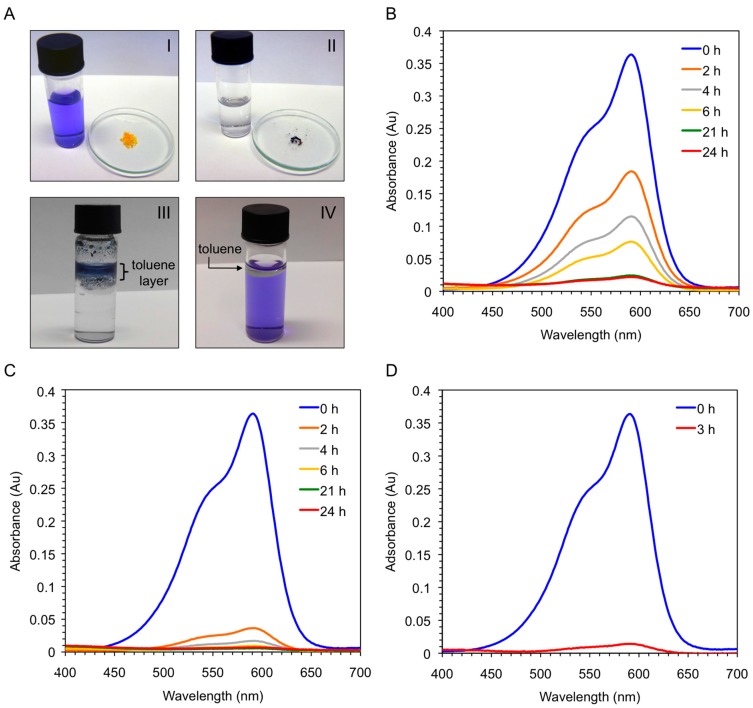
(**A-I**) Aqueous solution of crystal violet (*c* = 1 × 10^−5^ mol·L^−1^) before addition of either **bis-A4** (1.0% *w*/*v*; yellow powder in watch glass) or the corresponding xerogel obtained by freeze-drying the gel prepared from **bis-A4** in toluene (1.0% *w*/*v*); (**A-II**) Previous solution after dye removal by the xerogel (dark-blue powder in watch glass); (**A-III**) Mixture obtained after addition of toluene and **bis-A4** (1.0% *w*/*v*) to an aqueous solution of crystal violet (*c* = 1 × 10^−5^ mol·L^−1^). Ratio water:toluene 1:0.1 *v*/*v*. The picture was taken 3 h after shaking the mixture at room temperature; (**A-IV**) Mixture containing an aqueous solution of crystal violet (*c* = 1 × 10^−5^ mol·L^−1^) and toluene. Ratio water:toluene 1:0.1 *v*/*v*. The picture was taken after shaking the mixture at room temperature; (**B**) UV-vis absorption spectra of the aqueous solution of crystal violet showing the time-dependent adsorption of the dye by freshly synthesized peptide **bis-A4**; (**C**) UV-vis absorption spectra of the aqueous solution of crystal violet showing the time-dependent adsorption of the dye by the corresponding xerogel; and (**D**) UV-vis absorption spectra of the aqueous solution of crystal violet showing the time-dependent removal of the dye after addition of toluene (ratio water:toluene 1:0.1 *v*/*v*) and peptide **bis-A4** at room temperature.

Vey interestingly, the xerogel material obtained by freeze-drying the corresponding organogel was also able to uptake CV (>95%) within 6 h directly from the aqueous solution, whereas the freshly synthesized peptide needed one day to achieve the same result (Figure 9B,C). In principle, this result could be explained because the ability of the peptide to form supramolecular aggregates is considerably hampered in pure water, whereas the xerogel constitutes already a hydrophobic self-assembled network. Therefore, we decided to run a new experiment using the peptide and adding a small amount of toluene to the aqueous phase. Very interestingly, a quick migration of the peptide and the dye to the organic phase was observed at room temperature without the necessity of heating or sonicating the mixture. The kinetics of the dye removal was comparable to that using the xerogel material (Figure 9C,D), and the dye-containing organic layer could be separated from the clean aqueous phase by simple decantation. Notably, unless the organic phase is heated or sonicated the peptide remains insoluble in it without gel formation (Figure 9A-III). Moreover, simple addition of toluene to the dye-containing aqueous phase did not cause visible partition of the dye into the organic phase (Figure 9A-IV). Therefore, the peptide and/or peptide-dye complex is likely to adopt an energetically more favorable aggregate in the organic phase. However, further detailed experiments are still necessary in order to confirm this hypothesis and unequivocally determine the substrate specificity of the process and the exact nature of the intermolecular interactions between peptide and dye.

## 3. Experimental Section

### 3.1. Materials, Methods and General Procedures

#### 3.1.1. Materials

Unless otherwise stated all reagents and solvents were purchased from commercial sources (Sigma-Aldrich Chemie GmbH, Taufkirchen, Germany or TCI Deutschland GmbH, Eschborn, Germany) and used without further purification. All solvents were of analytical grade and water was bi-distilled prior to usage. Commercial xylene used in the experiments is a mixture of the three isomers. Gasoline and diesel fuel were purchased from gas outlets and used as received. River water was collected from the Danube River in Regensburg, Germany. The peptide-based gelators used in these studies were prepared as reported in our previous manuscript [38].

#### 3.1.2. Methods

^1^H NMR spectra were recorded at 25 °C on Avance 300 spectrometer (Bruker, Billerica, MA, USA) using D_2_O containing DMF (0.1 mmol) as internal standard. Ultrasound-induced PSG experiments were performed in a VWR™ ultrasonic cleaner (USC200TH, VWR International GmbH, Darmstadt, Germany). UV-vis spectra were recorded in a Varian Cary 50 UV-vis scanning spectrophotometer using 0.1 mm quartz cells (Suprasil^®^, Hellma, Müllheim, Germany). Oscillatory rheological measurements were made with an AR 2000 Advanced rheometer (TA Instruments, Eschborn, Germany) equipped with a Julabo C cooling system. A 1000 μm gap setting and a torque setting of 40,000 dynes/cm^2^ at 25 °C were used for the measurements in a plain-plate geometry (20 mm, stainless steel). The following experiments were carried out using 2 mL of total gel volume for the characterization of each sample: (1) Dynamic Strain Sweep (DSS): variation of *G'* and *G''* with strain (from 0.01% to 100%); (2) Dynamic Frequency Sweep (DFS): variation of *G'* and *G''* with frequency (from 0.1 to 10 Hz at 0.1% strain); (3) Dynamic Time Sweep (DTS): variation of *G'* and *G''* with time keeping the strain and frequency values constant and within the linear viscoelastic regime (strain = 0.1% strain; frequency = 1 Hz). Electron microscopy of the samples was conducted with the following instruments: (1) JEOL-2000 FXII transmission electron microscope (TEM, resolution = 0.28 nm, Jeol Ltd., Tokyo, Japan) equipped with a CCD Gatan 694 digital camera and operating at 10 kV (accelerating voltage); (2) Carl Zeiss Merlin™ field emission scanning electron microscope (FESEM, resolution 0.8 nm, Carl Zeiss, Germany) equipped with a digital camera and operating at 5 kV (accelerating voltage) and 10 μA (emission current). Preparation of the samples for TEM: 10 μL of the gel suspension was allowed to adsorb for 30 s onto carbon-coated grids (300 meshes, from TED PELLA, Inc., Plano GmbH, Wetzlar, Germany). After the adsorption, the excess solvent was removed by touching the edges with a small piece of filter paper (Whatman™, Sigma-Aldrich Chemie GmbH, Taufkirchen, Germany). The specimens were then dried overnight in a dessicator at low pressure and room temperature. Preparation of the samples for FESEM: Samples of the xerogels were prepared by the freeze-drying (FD) method: An Eppendorf tube containing the corresponding organogel (100–200 μL) was frozen in liquid nitrogen or dry ice/acetone and the sample was immediately evaporated under reduced pressure (0.6 mmHg) for 2 days at room temperature. The so-obtained fibrous solid was placed on top of a tin plate and shielded by Pt (40 mA during 30 s, film thickness ≈ 5 nm).

#### 3.1.3. General Procedure for Gel Formation

Typically, a weighted amount of the corresponding peptide and 0.5 mL of the appropriate solvent were placed into a screw-capped glass vial (4 cm length and 1 cm diameter) and gently heated with a heat gun until the solid material was completely dissolved. The resulting isotropic solution was then spontaneously cooled down to room temperature (RT). Note that no control over temperature rate during the heating-cooling process was applied during the preparation of the gels. The so-prepared material was preliminary classified as a “gel” if it did not exhibit gravitational flow upon turning the vial upside-down at room temperature. This state was further confirmed by oscillatory rheological measurements.

#### 3.1.4. General Procedure for Phase Selective Gelation (PSG)

Typically, a weighted amount of the corresponding peptide was added to a biphasic mixture of water (1.0 mL) and an appropriate organic solvent (0.5 mL), which was briefly warmed in order to dissolve the gelator or briefly treated in an ultrasound bath at room temperature (<60 s; extended ultrasound treatment could induce the formation of emulsions difficult to break with some mixtures). Selective gelation of the organic phase was achieved upon resting the sample at room temperature or *in situ* during sonication. Gelation was verified by complete absence of gravitational flow upon turning the vial upside-down. Alternatively, PSG can also be induced by adding a concentration solution (20%–40% *w*/*v*) of the peptidic gelator in the organic solvent to the corresponding oil/water mixture.

#### 3.1.5. Dye Adsorption Experiments

Typically, toluene (1 mL) and a weighted amount of peptidic gelator **bis-A4** (1.0% *w*/*v*) were added to an aqueous solution (1 mL) of crystal violet (*c* = 1 × 10^−5^ mol·L^−1^). The mixture was gently heated with a heat gun and subsequently cooled to room temperature. 

For the dye adsorption with xerogels, a weighted amount of the xerogel (prepared by freeze-drying the corresponding organogel prepared at 1.0% *w*/*v* in toluene) was added to an aqueous solution (3 mL) of crystal violet (*c* = 1 × 10^−5^ mol·L^−1^).

In a different experiment, toluene (0.3 mL) and a weighted amount of peptidic gelator **bis-A4** (1.0% *w*/*v*) were added to an aqueous solution (3 mL) of crystal violet (*c* = 1 × 10^−5^ mol·L^−1^). The mixture was shaken (150 rpm) at room temperature and the amount of dye transferred to the organic layer was determined using UV-vis spectroscopy. 

In each case, the amount of adsorbed dye over time was determined using UV-vis spectroscopy and a premade calibration curve. Concentration of initial dye was adjusted to avoid off-scale absorbance values.

#### 3.1.6. Determination of the Minimum Gelation Concentration (MGC)

MGC values were estimated by continuously adding solvent in several portions (50 μL each aliquot) into the vial where no gelation was achieved at the previous concentration and some material remained insoluble. The initial concentration used for gelation tests was 20% *w*/*v* (weight/volume, *i.e.*, 1% *w*/*v* = 1 g of solute per 100 mL of solution). The state of the new mixture was determined after the heating-cooling cycle as explained above. New experiments were made at lower concentration if stable gels were obtained at 20% *w*/*v*, and no further experiments were made if gelation was not achieved at 50% *w*/*v*. Non-gel containing vials (*i.e.*, clear solution after 1 day) were frequently left at room temperature and visually monitored for possible gelation and/or crystallization over time. Each measurement was made in duplicate and the average values are reported.

#### 3.1.7. Determination of the Thermal *Gel*-to-*Sol* Transition Temperature (*T*_gel_)

*T*_gel_ values were usually determined by the inverse flow method (IFM; *i.e.*, the seal vial containing the organogel was hung horizontally into an oil bath, which was heated up at 2 °C·min^−1^). Herein, the temperature at which the gel started to break was defined as *T*_gel_. Each measurement was made by duplicate and the average values are reported. The following procedure was used for materials derived from PSG: The aqueous phase after PSG was first removed from the vial using a syringe. The remaining gel-body was then melted applying gentle heat and left for cooling at RT. After reformation of stable gel-materials, *T*_gel_ values were determined using the IFM as described above. The results obtained with this method have been previously well correlated with the first endothermic transition observed by modulated differential scanning calorimetry (DSC).

## 4. Conclusions

In conclusion, the PSG of organic phases from their non-miscible mixtures with water can be achieved using tetrapeptides bearing a side-chain azobenzene moiety. The presence of the azobenzene unit is necessary to achieve PSG at the same concentration than the MGC necessary to obtain gels in pure organic solvents, which are very similar in terms of thermal, mechanical, and morphological properties to those obtained by PSG. However, when miscible oil/water mixtures were used, the entire mixture was gelled even at lower concentration than the MGC determined for the pure organic phase. Importantly from a practical point of view, PSG can be triggered at room temperature by ultrasound treatment of the mixture or by adding an ultrasound-aided concentrated solution of the peptide in an oil-phase to a mixture of the same oil and water. In addition, the process is not affected by the presence of salts or impurities existing in natural water, it can be scaled-up, and both the oil phases and the intact gelator can be recovered almost quantitatively after simple distillation. These peptidic gelators and their PSG ability can be also used to quantitatively remove toxic dyes from aqueous solutions within minutes. Finally, it should be mentioned that preliminary experiments have shown that PSG could also be attained using analog peptides bearing unprotected amino termini [39]. This offers another interesting possibility for uptake and delivery of bioactive guest molecules in a controlled manner from one phase into another by coupling PSG with *trans*-to-*cis* photoisomerization of the chromophore that leads to *gel*-to-*sol* transition, which requires an unprotected amino terminus [38]. Research in this direction is currently ongoing in our laboratory.
